# The Health Physician’s Fourth Annual Report of the Mortality of the City of Buffalo for 1857

**Published:** 1858-04

**Authors:** 


*The Health Physician's Fourth Annual Report of the Mortality of the City of Buffalo for* 1857.

We have delayed for a little our notice of Dr. Dayton's brief and comprehensive report, that we might give it such a notice as the importance of the subject on which it touches demands. In the year 1854 the Board of Health was organized as usual, but with new men in it control. A. F. Lee was President of the Board, and James M. Newman, physician. Up to that time the records of deaths had been imperfect, and the action of the Board of a spasmodic or intermitting character, the sanitary laws being enforced only in epidemic seasons, and allowed to lie dormant in intervals of health. The Board was not what it should be, a constant, ever-acting power, always carefully watching the inroads of disease, or engaged in increasing our chances of health. But in 1854 a new regimé was set up. The sanitary laws were made operative, and the business of the Health Office reduced to system and efficiency. Under all subsequent officers the system inaugurated by Dr. Newman had been maintained, and we believe that no department of our city government has been more faithfully administered than this. The present Board is well made up, and will sustain the reputation of the office.

During the year 1857 there have been 2,268 deaths from all causes. This, assuming the population to be 100,000, would give a ratio of mortality of *one in forty-four*, indicative of great good health in our community. But it should not be forgotten that disease is not the only cause of death, and that a careful analysis of Dr. Dayton's tables would show that a very considerable deduction from our mortality proper may be made. It would be an interesting study to take this 2,268 deaths, and deduct from it all which are not the result of disease, of if of disease, such as are clearly preventable. Running over the tables, we can segregate the following list of this class: Small pox, 2; syphilis, 6; delirium tremens, 3; accidents by steam explosions, etc., 42; burned, 2; drowned, 38; fractures, 5; intemperance, 23; murder, 1; poison, 2; suffocation, 2; suicide, 6; still-born, 90. These foot up a total of 222, which, deducted from the grand total, leave us only 2,046 deaths from what may be considered as unavoidable diseases.

But are all these unavoidable? We do not mean to apply the strict physiological idea that all deaths are the result of violations of the laws of nature except those from old age, but simply to still further segregate a class of diseases are in themselves of such a nature as to be prevented if we knew how, or if knowing how, we would do it. Who believes that it was "by visitation of God and not otherwise," that 1,434 little children under five years of age have died in this city during the past twelve months? Who does not know that a very large proportion of the 213 deaths from convulsions, the 126 from cholera infantum, the 94 from diarrhœa, and the 57 from dysentery might have been avoided by a proper attention to diet, cleanliness, ventilation and exercise on the part of those having control over them?

There is an element of responsibility involved in this preventability of disease which is not so fully appreciated as it might be. The children of the rich stand twice the chance of life that is given the children of the poor. Take a class of one hundred new-born children on the Points, the flats, or any other district where want and dirt are combined—half will be dead before the end of a year, and not one quarter will ever see five years of age. With the rich it is different, and though the ratio of infantile deaths among the comfortable classes might be hard to get at, yet the data exist for an approximate estimate. The liabilities of childhood in an ordinary season correspond very closely with those of the adult in an epidemic season. In the fall of 1854 we published a statement, which showed, very conclusively, that the blessings of health were largely dependant on our household comforts and out-door conveniences, such as good pavements and sewers, fresh air and pure water. A comparison was made between a district bounded east and north by the city line, south by Clinton street, and west by Michigan street, in which, during the months of June, July, August and September, 1854, died 341 souls from cholera and its allied diseases, while a corresponding district, bounded north by the city line, east by Main street, west by Delaware, and south by the Terrace, had during the entire disastrous season of 1856, only 22 deaths from the same class of disease. A similar comparison of the number of cases of cholera on paved or unpaved streets presented results equally startling.

Thus cholera—the black tiger of India—is a preventable disease. And the same causes which disarm it of its terrors are equally efficient in the whole class of epidemics, and in many cases of the epidemic diseases. The lesson is encouraging, for it teaches us that sanitary precaution is not in vain, and that an efficient health-police may throw a bulwark around the life of the citizen.

